# The relationship between maternal-fetus attachment and perceived parental bonds in pregnant women: Considering a possible mediating role of psychological distress

**DOI:** 10.3389/fpsyg.2022.1095030

**Published:** 2023-01-16

**Authors:** Maria C. Gioia, Antonio Cerasa, Vito M. R. Muggeo, Paolo Tonin, Juanita Cajiao, Alessia Aloi, Iolanda Martino, Flaviana Tenuta, Angela Costabile, Francesco Craig

**Affiliations:** ^1^iGreco Ospedali Riuniti, Cosenza, Italy; ^2^Associazione di Volontariato Mammachemamme, Cosenza, Italy; ^3^Institute for Biomedical Research and Innovation (IRIB), National Research Council of Italy (CNR), Messina, Italy; ^4^Pharmacotechnology Documentation and Transfer Unit, Preclinical and Translational Pharmacology, Department of Pharmacy, Health Science and Nutrition, University of Calabria, Rende, Italy; ^5^S’Anna Institute, Crotone, Italy; ^6^Department of Economics, Business and Statistics, University of Palermo, Palermo, Italy; ^7^Faculty of Medicine Universitat de Barcelona, Barcelona, Spain; ^8^Department of Medical and Surgical Sciences, Institutes of Neurology, Magna Græcia University of Catanzaro, Catanzaro, Italy; ^9^Department of Cultures, Education and Society, University of Calabria, Cosenza, Italy; ^10^Scientific Institute, IRCCS E. Medea—Unit for Severe Disabilities in Developmental Age and Young Adults, Brindisi, Italy

**Keywords:** prenatal attachment, maternal-fetal attachment, parental bonding, maternal care, paternal care

## Abstract

Maternal-Fetal Attachment (MFA) delineates the emotional, cognitive, and behavioral aspects that mothers develop toward the unborn baby during pregnancy. The literature indicates that optimal attachment in pregnancy represents a protective factor for the mother-child attachment bond after birth and child development outcomes. To date, there are few studies that have investigated associated factors of MFA. This study sets out to explore the association between perceived parental bonds and maternal-fetal bonding in pregnant women, accounting for factors such as psychological distress, socio-demographic and obstetric characteristics.

**Methods:**

In this cross-sectional study, 1,177 pregnant women answered the Parental Bonding Instrument, the Maternal-Fetal Attachment Scale, State-Trait Anxiety Inventory (STAI-Y), and Beck-Depression Inventory (BDI-II).

**Results:**

We found out that perceived maternal and paternal care had significant direct effects on maternal-infant bonding during the pregnancy period when controlling for some confounders, including gestational age and mother age among others. Such maternal and paternal perceived care effects were not mediated by levels of psychological distress, which in turn resulted to be a “borderline” significant predictor of prenatal attachment. Interestingly, the gestational age and the mother age emerged to have a significant and synergic nonlinear effect, suggesting the influence on the MFAS of the gestational age depends on the values of the mother age, and likewise, the effect of mother age on MFAS depends on the gestational week.

**Conclusion:**

This study expands our knowledge of the intergenerational transmission of attachment pointing out the effects of a woman’s perceived bond in relation to her parental figures during the development of the prenatal attachment process. Findings also suggests that parenting support interventions may have benefits that are realized across generations.

## Introduction

The experience of pregnancy has a strong impact on women’s lives. It is a unique period that might produce specific difficulties for the future mothers, adapting to this challenge through positive and negative reactions (Walsh, 2010; Su et al., 2015; Copeland and Harbaugh, 2019). Considering the importance of the child’s representation in the early developmental stages of pregnancies, some authors have studied the internal emotional representation of the future newborn and the maternal-fetus prenatal relationship as a unique experience (Atashi et al., 2018). Some authors have used the term “maternal–fetal attachment” (MFA) to define the emotional attachment between the mother and fetus as an indicator of their health and the mother’s efficiency in the postnatal period (Ckanley, 1981; Alhusen, 2008). Similarly, some researchers used the term “prenatal attachment”, referring to an emotional relationship that parents develop toward the unborn baby during the gestation period (Brandon et al., 2009; Atashi et al., 2018). The extensive literature on prenatal attachment has demonstrated that maternal prenatal attachment contributes to structuring the perception of the child as a human being and future developments of the attachment between the mother and the baby (Akbarzadeh et al., 2016; McNamara et al., 2019; Howland et al., 2020). Through this relationship, during the gestation period the woman expresses to the fetus concerns, affections, and expectations, influencing the postnatal maternal-child attachment and the adequacy of the care (McNamara et al., 2019; Salehi et al., 2019; Howland et al., 2020). In a recent systematic review of 41 studies, Rollè et al. (2020) reported that poor prenatal attachment is linked with low prenatal maternal fetus representations and self-care, low postnatal attachment until 24 months postpartum and early child development problems such as emotional self-regulation, behavioral and cognitive difficulties (Alhusen et al., 2012b; Maddahi et al., 2016; Rossen et al., 2016; Daglar and Nur, 2018; Arguz Cildir et al., 2020). Therefore, it is important to be aware of the factors that may have an influence on a prenatal attachment process and attachment-related behavior.

A key element that might be associated with prenatal attachment is the woman’s perceived bond in relation to own parental figures during her childhood and adolescence, denominated parental bond. Bowlby states that the mother’s psychological state during parenting is related to “a long history of interpersonal relations within her family of origin” (Bowlby, 1982), suggesting that parental bonding is the perception of established bonds that a person has toward his/her parents. Therefore, the parenting of grandparents could regulate the behaviors and emotions of mothers toward their children. These attitudes in future mothers are associated with better quality of maternal-fetus prenatal attachment since positive past parental behaviors contribute to consolidating levels of care and protection and positive interactions between the mother and her fetus (Handelzalts et al., 2018). Care and protection are two important components in child-rearing and adequate parenting is attributed to high care and low protection (Parker, 1990). One study by Reed (2014) suggests that the combination of high parental overprotection and low care during a woman’s childhood presents a risk factor for weakened prenatal attachment levels and potentially low levels of attachment after birth. In contrast, a previous study detected a positive correlation between paternal overprotection and the intensity of maternal concern, particularly in the second gestational trimester (Van Bussel et al., 2010). da Rosa and colleagues, in a recent large-sample longitudinal study, detected the importance of the father’s role in the women’s life, showing that the gestational women who perceived paternal overprotection during their development reported better prenatal attachment (da Rosa et al., 2021). Thus, empirical data regarding the potential relationship between perceived parental bonding and the relationship between a pregnant woman and her fetus are scarce and inconclusive. Moreover, the literature shows that the quality of the mother’s attachment and maternal behaviors to her fetus has important implications in the postnatal period, influencing the postnatal attachment and the adequacy of the care (Tambelli et al., 2014; Ana Luisa et al., 2019; McNamara et al., 2019; Howland et al., 2020; Lutkiewicz and Bidzan, 2022). Considering the above mentioned findings, it is evident the importance of identifying associated factors of the prenatal attachment process, such as a parental bond, since it is implicated with negative outcomes for the infant-mother dyad.

Furthermore, during the transition to parenthood, which is a time of intense psychological change, women are also at risk for the onset of psychological distress. The World Health Organization reports that about 10% of pregnant women experience psychological distress, especially anxiety and depression that generally leads to adverse outcomes for early mother-child bonding (WHO). Prenatal anxiety, depression, and stress can result in pregnancy complications, as well as difficulties in the establishment of the mother-infant bond, which adversely affects the pregnant women’s attachment process, which is essential to the baby’s psychological development. Some authors suggested that psychological distress may compromise capacity to feel adequate in her new role as mother or be related with a general state of detachment that also affects her ability to bond with the fetus (Isgut et al., 2017; Tokuda et al., 2021). Additionally, a noteworthy number of studies have looked at the relationship between psychological distress and parental bonding, showing that the clinical population reported lower parental care than the general population (Røhder et al., 2020). Previous studies, using the Parental Bonding Instrument (PBI), showed that poor parenting styles, characterized by “low care” or “high overprotection” are also associated with anxiety and depressive symptoms in women in the perinatal period (McMahon et al., 2005; Fukui et al., 2021). So far, however, the associations between prenatal parental reflective functioning, psychological distress, and specific dimensions and risk profiles of maternal-fetal bonding in pregnant women have not been closely examined. It has been reported that several socio-demographic and obstetric characteristics such as socioeconomic status, pregnancy age, education level, unplanned pregnancy, being primigravida, social support quality and quantity, may be associated with worse MFA or onset of bonding disorder (Alhusen et al., 2012a; Jamshidimanesh et al., 2013; Lau et al., 2018; Sacchi et al., 2021). Thus, the identification of several risk factors in this regard is a pressing research topic.

**FIGURE 1:**
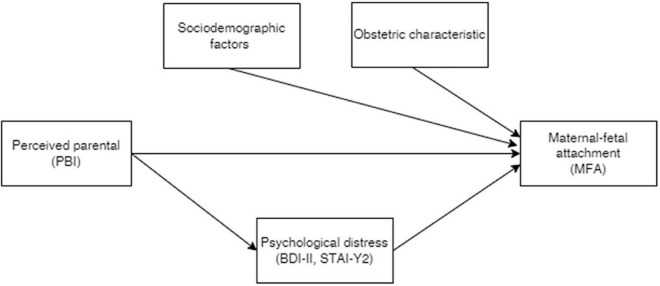
The conjectured relationships between the response maternal–fetal attachment (MFA), parental bonding instrument (PBI), and psychological distress.

The study’s aim is to examine how parental bonding and psychological distress are associated with maternal fetal attachment in a broad sample of pregnant women. Our pre-analyses conjectures are reported in Figure 1: first, parental perceived bonds could predict maternal–fetal attachment quality in the perinatal period; second, psychological distress would mediate the relationship between parental bonding and maternal–fetal attachment. Other socio-demographic and obstetric characteristics have been evaluated as possible confounders.

## Materials and methods

### Participants

The study was authorized by the University of Calabria Ethics Committee (Protocol number: 0013005). The study was conducted in accordance with the Declaration of Helsinki. Data collection took place from 14 March and 25 April 2020 among pregnant Italian women. The study was conducted through an anonymous, web-based, cross-sectional survey developed with Google Forms, and was supported by two local non-governmental associations, the “Associazione di Volontariato Mammachemamme” and the “Movimento di Psicologia Perinatale”. The survey’s URL was sent to the participants through social media, emails, and advertisements in Italian prenatal and natal clinics. The inclusion criteria were as follows: willing to participate, having reading skills and language comprehension to complete the self-report questionnaire, no history of mental disorders or substance abuse and drug addiction. The intent of the study was explained to the participants, who gave formal consent and did not receive any reward.

### Measures

A questionnaire containing items about the mother’s age, civil status, educational level, disease history, number of children, gestational age, history of abortion, pregnancy complication was used to collect the socio-demographic and obstetrics data.

Maternal–fetal attachment in pregnant women were measured through the Maternal–Fetal Attachment Scale (MFAS) (Ckanley, 1981). The MFAS is a 24-item self-report questionnaire developed to measure the extent of the attachment between a mother and her unborn child. Each item is scored from 1 (Definitely No) to 5 (Definitely yes). The total score ranges from 24 to 120 and higher scores indicate greater attachment between mother and fetus. Negative items namely item numbers 9 and 24 will be reverse scored. MFAS contains five subscales: Differentiation of self; Interaction with the fetus; Attributing characteristics and intentions; Giving of Self; Role taking. The reliability (internal consistency) of the scale was assessed and reported as α = 0.85 by its developer (Ckanley, 1981). Busonera and colleagues (Busonera et al., 2016) evaluated the validity and reliability of the Italian version of this tool and calculated Cronbach α coefficient. The total MFAS scale showed a good internal consistency, with α of 0.77. Item–scale correlations ranged from 0.22 to 0.44.

Prenatal bonding was measured through the PBI (Parker, 1990). The PBI is a 25-item self-report measure of two parenting styles, “Care” and “Overprotection/Control.” The instrument assesses these self-perceived relationships separately for the mother and the father. The care dimension measures positive parenting, including parental warmth and affection. The Control dimension measures negative parenting, including parental control and constraint. We chose the PBI because it is a widely used tool for examining the relationship between parental attitudes and mental-physical health in different populations such as pregnant women. A test-retest reliability study yielded a Pearson correlation coefficient for the care scale of 0.761 and a Pearson correlation coefficient for the overprotection scale of 0.628 (Parker, 1990).

To estimate levels of state and trait anxiety, the survey used is the State-Trait Anxiety Inventory (STAI-Y) (Spielberger and Sydeman, 1994). The items investigating state anxiety are 20 (STAI Form Y-1), exploring feelings of tension, anxiety, and nervousness. Trait anxiety levels are explored with the 20 items of the trait anxiety scale (STAI FORM Y-2), estimating anxiety levels, with a four-point Likert scale. For both scales, scores ranged from a minimum of 20 to a maximum of 80. Mild anxiety is indicated by a cutoff score of 40–50, while 50–60 indicates moderate anxiety, and over 60 indicates severe anxiety. Internal consistency of the scale ranges from 0.86 to 0.95; test-retest reliability ranges from 0.65 to 0.75 in a 2-month interval. In the present study, test-retest coefficients ranged from 0.60 to 0.89.

Depression levels were measured through the Beck-Depression Inventory (BDI-II), consisting of 21 self-report items (Beck et al., 1996). This scale is very effective for the assessment of depression symptoms and of their severity in the population. The items have a rating from zero to three and are summed to obtain a global score of 0–63, with higher scores associated to higher levels of depression. In the range 0–13 the score indicates minimal depression, 14–19 mild depression, 20–28 moderate depression, and 29–63 severe depression. Internal consistency of the test was around 0.92 and test-retest reliability is 0.93.

### Statistical methods

Symmetric relationships among numeric variables were investigated by means of the Pearson correlation coefficients. The parental perceived bonds are represented by two pairs of variables for both mother and father, namely: the maternal/paternal perceived care (MPC and PPC) and the maternal/paternal perceived over-protection (MPO and PPO). To quantify their effects on the maternal-fetus attachment (MFAS), we ran a regression model for the expected value of MFAS as a function of MPC, PPC, MPO, and PPO, also including additional variables such as mother age, previous childbirth, gestational age and psychological distress (STAI-2 and BDI). Moreover, to assess if the parental bonds effects on MFAS were also partially “mediated” by the psychological distress, specifically the STAI-2 and BDI-II variables, we fitted a second regression model having the STAI-2 and BDI-II as a response and the same aforementioned factors as covariates. Taking the two fitted regression models as input, we carried out mediation analysis (Mackinnon, 2012) to estimate the average direct effects (ADE) and the average conditional mediated effects (ACME).

The statistically significant *P*-value was set at 0.05. All statistical analyses were done using the software R (version 4.0.5) and packages mediation (Imai et al., 2010) and betareg (Cribari-Neto and Zeileis, 2010).

## Results

Table 1 shows the socio-demographic, obstetric, and clinical characteristics of the participants of the *n* = 1,179 pregnant women enrolled in the study. The age ranging from 18 to 56 years (32.83 ± 4.3 years), and the mean gestational age ranging from 5 to 41 weeks 26.28 ± 8.9. Most of the participants were married (63,2%) and nearly half of them had University degrees (46.6%). With respect to pregnancy aspects, the 42,7% had previous pregnancies, the 26,6% reported history of abortion, and the 13,2% had pregnancy complication.

**TABLE 1:** Clinical, sociodemographic, and obstetric characteristics of the pregnant women.

	*N* (1,177)	%
**BDI**		
Minimimal (<14)	746	63
Mild [14–19]	252	21.4
Moderate [20–28]	146	12.4
Severe [29+]	33	2.8
**STAI Form Y-2**		
Minimal	470	39.9
Mild	406	34.5
Moderate	237	20.1
Severe	64	5.5
**Educational level**		
Secondary school	56	4.8
High school	374	31.7
University	549	46.6
Post university	198	16.9
**Civil status**		
Married	744	63.2
Single	49	4.2
Divorced/separated	17	1.4
Cohabiters	367	31.2
**Previous children**	558	47.3
**History of abortion**	314	26.6
**Pregnancy complication**	156	13.2
**Maternal age (range 18–56 years)**	32.8 (4.3)	
**Weeks of gestation (range 5–41 weeks)**	26.3 (8.9)	
	**Mean**	**Sd**
**MFAS total (range 61–113)**	91.8	8.44
Differentiation of self (range 9–20)	16.6	2.2
Interaction with the fetus (range 11–25)	19.2	2.73
Attributing characteristics and intentions (range 11–29)	20.4	2.84
Giving of self (range 11–25)	17.9	2.34
Role taking (range 8–20)	17.7	2.31
**PBI**		
Maternal care (range 0–63)	25.4	7.66
Paternal care (range 0–36)	21.9	9.05
Maternal control (range 0–38)	13.9	7.22
Paternal control (range 0–38)	12.5	7.38
**BDI (range 0–54)**	12.4	7.39
**STAI Form Y-2 (range 21–79)**	43.9	9.91

PAI, Prenatal Attachment Inventory; BDI-II, Beck Depression Inventory; STAI-Y, State-Trait Anxiety Inventory.

The mean total score on the MFAS was 91.8, and the mean subscale score were: 16.6 (Differentiation of self), 19.2 (Interaction with the fetus), 20.4 (Attributing characteristics and intentions), 17.9 (Giving of Self), 17.7 (Role taking). The mean total score for STAI Form Y-2 and BDI-II was 43.9 and 12.4, respectively. 63.4% of women had no depressive symptoms, 18.9% had mild depressive symptoms, 14.2% had moderate symptoms and only 3.5% severe symptoms. On the Trait-Anxiety scores, 23.2% had mild trait anxiety levels, 31.1% had moderate levels and 28% had severe levels. Among the anxiety levels, out of 1,179 women 36.6% had mild state anxiety levels, 20.4% had moderate levels, and 7% had severe levels.

We fit a regression model including the MFAS score as the outcome variable and the parental bonds scores, namely MPC, MPO, PPC, and PPO. Also, socio-demographic/obstetric characteristics, depression symptoms, and trait anxiety were entered simultaneously as explanatory variables. Results reported in Table 2 indicated that both maternal (*p* = 0.022) and paternal (*p* = 0.004) perceived care is significatively and positively associated with MFAS, sharing a somewhat similar effect on the response; on the other hand, no significant effect was found for the overprotection scores, i.e., MPO and PPO. Moreover, we found no evidence against linearity in the effects of MPC/MPO and PPC/PPO on the MFAS and also no relevant interaction terms between such variables were deemed to be significant, suggesting no synergic effect on the response.

**TABLE 2:** Parameter estimates for the outcome regression model having Maternal–Fetal Attachment Scale (MFAS) a response.

Predictors	Estimates	Std. Error	95% CI	*P*-value
MPC	0.079	0.035	0.011–0.147	0.022*
MPO	0.031	0.037	−0.041 to 0.104	0.400
PPC	0.081	0.028	0.026–0.137	0.004*
PPO	0.030	0.035	−0.039 to 0.098	0.398
BDI-II-total score	0.070	0.045	−0.019 to 0.159	0.125
STAI form Y2	−0.064	0.034	−0.131 to 0.002	0.059
Previous children (Yes vs. No)	−2.636	0.480	−3.577 to −1.694	<0.001**
gest age [log]	5.600	0.548	4.525–6.675	<0.001**
age [1st degree]	−55.00	59.96	−172.6 to 62.63	0.359
age [2nd degree]	−113.5	61.30	−233.8 to 6.698	0.064
gest age [log]^†^ age [1^st^ degree]	11.20	18.57	−25.18 to 47.68	0.545
gest age [log]^†^ age [1^st^ degree]	40.50	18.93		0.032*

PAI, Prenatal Attachment Inventory; BDI-II, Beck Depression Inventory; STAI-Y, State-Trait Anxiety Inventory; MPC, maternal perceived care; PPC, paternal perceived care; MPO, maternal perceived overprotection; PPO, paternal perceived overprotection. *p-value < 0.05. **p-value < 0.01. ^†^Means product between the two terms to account for the interaction.

Significant effects on the fetus attachment were also obtained for the “previous childbirth” and the psychological distress as measured by trait anxiety (“STAI-form Y2”) exhibiting a somewhat borderline but noteworthy *p*-value (*p* = 0.059). Depression level (BDI-II score) appears not to be related to the fetus stimulus. To investigate the mediating role of STAI-form Y2 in the relationships between MFAS and MPC/PPC we ran a mediation analysis by fitting the “mediator” regression model having the STAI-form-2 as response and the same covariates of the outcome model (Table 3): results of mediation analysis suggest that the effect of parental perceived cares, both MPC and PPC, on MFAS are entirely direct with no mediating role of STAI form 2.

**TABLE 3:** Parameter estimates for the mediator regression model having State-Trait Anxiety Inventory (STAI) form Y2 as a response.

	STAI-Y			
**Predictors**	**Estimates**	**Std. Error**	**95% CI**	***P*-value**
MPC	−0.053	0.030	−0.111 to 0.006	0.078
MPO	0.031	0.032	−0.032 to 0.094	0.332
PPC	−0.038	0.024	−0.085 to 0.010	0.122
PPO	0.003	0.030	−0.056 to 0.062	0.916
BDI-II-total score	0.938	0.028	0.882–0.993	<0.001**
Previous children	0.989	0.414	0.178–1.801	0.017*
gest age [log]	−0.411	0.473	−1.340 to 0.517	0.385
age [1st degree]	−92.28	51.72	−193.7 to 9.190	0.075
age [2nd degree]	50.92	52.92	−52.91 to 154.7	0.336
gest age [log]^†^ age [1st degree]	24.52	16.02	−6.908 to 55.96	0.126
gest age [log]^†^ age [2nd degree]	−17.13	16.34	−49.19 to 14.92	0.295

PAI, Prenatal Attachment Inventory; BDI-II, Beck Depression Inventory; STAI-Y, State-Trait Anxiety Inventory; MPC, maternal perceived care; PPC, paternal perceived care; MPO, maternal perceived overprotection; PPO, paternal perceived overprotection. Observations 1,177. R^2^/R^2^ adjusted. 0.538/0.534. *p-value < 0.05. **p-value < 0.01. ^†^Means product between the two terms to account for the interaction.

We found strong evidence that both MPC and PPC have a significant effect on fetus attachment. As a sensitivity analysis, we also checked for possible non-linear and synergic effects, but no evidence was found.

In addition, we repeated the regression analysis with all five MFAS subscales as outcome variables. Results showed that MPC is a significant predictor of Role Taking (*p* = 0.038), Differentiation of self from fetus (*p* = 0.044), and Interaction with fetus (*p* = 0.009); while PPC is a significant predictor of Role Taking (*p* = < 0.001) and Attributing characteristics to fetus (*p* = 0.024). Results are summarized in Table 4.

**TABLE 4:** Estimated total effect, relevant *p*-value and proportion of the effect mediated by STAI2 for the variables maternal perceived care (MPC) and paternal perceived care (PPC) on different outcomes.

	Est	*P*-value	%mediated
**MPC**			
Differentiation of self	0.018	0.044*	8.10%
Interaction with the fetus	0.031	0.009**	4.90%
Attributing characteristics and intentions	0.005	0.59	0.10%
Giving of self	0.006	0.52	0.15%
Role taking	0.021	0.038*	4.10%
**PPC**			
Differentiation of self	0.014	0.079	8.20%
Interaction with the fetus	0.016	0.064	0.07%
Attributing characteristics and intentions	0.021	0.024*	0.01%
Giving of self	0.007	0.4	1.30%
Role taking	0.028	<0.001**	2.40%

MPC, Maternal perceived care; PPC, paternal perceived care. *p-value < 0.05.

**p-value < 0.01.

**FIGURE 2:**
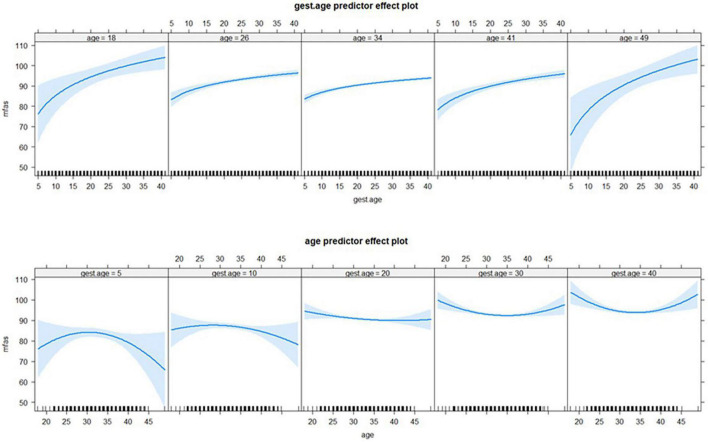
The synergic non-linear effect of mother age and gestational age on MFAS: The **top** panels portray the effect of gestational age by different values of mother age (18, 26, 34, 41, and 49 years). Specularly, the **bottom** panels show the effect of mother age by specific values of gestational age i.e., at 5, 10, 20, 30, and 40 weeks.

Interestingly, the gestational age and the mother age emerged to have a significant and synergic non-linear effect, suggesting the influence on the MFAS of the gestational age depends on the values of the mother age, and likewise, the effect of mother age depends on the gestational week. Non-linearity in both covariates was modeled *via* a quadratic polynomial (for the mother age) and the log transformation for the gestational age. Figure 2 depicts such non-linear and synergic effects.

## Discussion

The objective of this study was to evaluate the effects of perceived parental bonding on maternal prenatal attachment, after controlling for relevant psychological distress symptoms, and socio-demographic and obstetric variables. The main findings revealed that perceived maternal and paternal care both had significant direct effects on maternal–infant bonding during the pregnancy period, indicating that the pregnant women who perceived high parental care during their childhood and adolescence had positive maternal-fetal relationships. Psychological distress was considered as the observed variable that acts as a mediator in the model, however, the effect of parental care on MFA is not mediated by levels of anxiety or depression. Further, no significant relations were found between the perceived maternal/paternal control and MFAS scores. These findings accord with that of Fukui et al. (2021) who found that perceived care for maternal and paternal parenting is a predictor of the attachment process during the pregnancy period. However, the current result is not unexpected given that positive parental or caregivers care represents a protective factor for child cognitive-emotional-social development, and resilience and self-esteem in adulthood (Enns et al., 2002; Tian et al., 2018; Sameshima et al., 2021); while excessive parental control or intrusive behavior hinders the ability to be independent and increases the risk of insecure attachments, and emotional and behavioral problems (Festa and Ginsburg, 2011; Sameshima et al., 2021; Kidd et al., 2022). Hence, the current result suggests that women who receive adequate care from their father or mother before adolescence establish sufficient bonding with their infants in the perinatal period. This is in line with the developmental perspective of intergenerational transmission of parenting style, indicating continuity in the style across generations (Van Ijzendoorn, 1992). Intergenerational transmission is considered the influence of parents’ own experiences as a child on their later childrearing practices (Jarnecke and South, 2013; de Cock et al., 2016; Taccini et al., 2021). Although the majority of studies have focused on the intergenerational continuity of trauma transmission or aggressive-intrusive parenting (Belsky et al., 2009; Ahlfs-Dunn et al., 2022), there is a body of research suggesting such transmission exists also true for warm and supportive parenting (Kretchmar and Jacobvitz, 2002; Madden et al., 2015). Intergenerational transmission of parenting can be explained by genetic and environmental factors (Branje et al., 2020; Craig et al., 2021). First, recent developments in epigenetic research have led to a growing interest in the effects of parental care on the role of biological mechanisms (e.g., DNA methylation) in mediating environmentally induced changes in parental care that are transmitted across generations (Champagne, 2008; Montirosso et al., 2020; Craig et al., 2021). Hence, understanding whether the relationship between perceived parental care and maternal-fetal attachment would be mediated by epigenetic mechanisms could be a key issue in future studies. Second, a direct mechanism is a child observes the behavior of his/her parents and reproduces this parental style when becoming a parent or a child develops an attachment style because of parent-infant interaction, which is replicated when the child becomes a parent (Madden et al., 2015), probably already during the pregnancy period when the prenatal attachment is developing. In support of this possibility, we have found a significant direct effect of perceived both maternal and paternal bonding on the MFAS subscales, in particular the role-taking scale, attributing characteristics to fetus, differentiation of self from fetus, and interaction with fetus. These findings suggest that women who receive adequate care from their father or mother reported mostly positive feelings toward their pregnancy with greater pregnancy acceptability, and they can more easily establish a relationship with their fetus and attribute emotions or behaviors to it. Adjusting to parenthood during pregnancy brings with it lots of biological, physical, and psychological changes that make them particularly vulnerable to psychological distress. Thus, high perceived parental care could be a protective factor for adjusting to the idea of pregnancy and motherhood leading to a decrease in distress, and better antenatal bonding. However, this hypothesis must be interpreted with caution because of several confounding factors not included in this study such as the role of gender, relationship quality, and social support (della Vedova et al., 2019; Göbel et al., 2019; Sacchi et al., 2021).

Another important finding was that state anxiety resulted to be a “borderline” significant predictor of prenatal attachment after adjusting for the other socio-demographic and obstetric information. We found that women who have a higher state of anxiety may experience a lower quality of maternal-fetal attachment. In accordance with the present results, previous studies have demonstrated that anxiety is one of the strongest predictors of poor psychological well-being in pregnant women and of lower quality of MFA (Allison et al., 2011; Mazzeschi et al., 2015; Napoli et al., 2020). It is possible, that elevated state anxiety during pregnancy influences the quality of prenatal attachment, meaning less time on attachment-related behavior (e.g., palpating, talking, and thinking to the fetus) or may result in less pre-occupation with the pregnancy and the fetus (i.e., as reflected in lower maternal-fetal attachment scores). Thus, the results from our study expand prior findings by suggesting that pregnant women who experienced anxiety during pregnancy may be at an elevated risk of reporting a decreased amount of interaction with their fetus (Shahhosseini et al., 2015; Napoli et al., 2020). In contrast to expectations (Rollè et al., 2020), this study did not find a significant association between depression and MFAS scores. However, it is necessary to interpret these results with caution due to the many studies that did not confirm these results. The absence of a significant association, also in the current study, could be due to methodological issues such as assessment time, type of target population or confounding variables that were controlled for and that consequently hid the effect of prenatal depression symptoms on prenatal attachment (e.g., disease history, number of children, gestational age, history of abortion, pregnancy complication).

In the multivariable analysis, gestational age and the age of the woman appear to influence prenatal attachment. As expected, gestational age showed a positive association with MFA. These results corroborate the findings of a great deal of the previous works. It has been demonstrated that prenatal attachment behaviors increase with advancing gestational age and the latter is associated with a positive effect on the fetus such as thinking about her involvement in the dyadic interaction, and interaction and maternal planning (Barone et al., 2014). Hence, it is possible to assume that the women can perceive the new movements of the baby during pregnancy, which makes the experience more corporeal for them and may lead them to interact more adequately with the fetus. In addition, previous studies also detected that MFA intensity may be affected by factors such as maternal age (Čėsnaitė et al., 2019; McNamara et al., 2019). Pregnant women who were younger than 20 years shows the lowest levels of prenatal attachment compared to women who were older (Ossa et al., 2012; Canlı and Demirtaş, 2022). It may be that younger women may experience ambiguous and mixed feelings about the physical and psychological changes during pregnancy and may not feel ready to be mothers (Crozier et al., 2009; Wilson-Mitchell et al., 2014), all of which may blunt their prenatal attachment levels. In contrast, other studies detected no association between participants’ ages and prenatal attachment (Daglar and Nur, 2018; Ulu and Bayraktar, 2018). We found that the relationship between MFA and the mother’s age is quadratic. Prenatal attachment is greater for both younger and older women and reaches a minimum of around 34 years of age. Interestingly, the finding showed the synergic non-linear effect of mother age and gestational age on MFAS. The maternal prenatal attachment is higher for women around 30–35 years old in the early weeks (within 10, say) of the gestational period, but in the late gestational period (after 30 weeks, say) the relationship is the opposite and the maternal prenatal attachment is higher for younger or older women rather than the middle women aged 30–35 years. Most of the studies considered a diverse range of individual and obstetrics variables as potential risk or protective factors, but no one has yet evaluated the interaction between them. Elucidating the role of a combination of the mother’s age together with gestational age in a prenatal attachment will be a new direction for future studies.

## Limitations

Some limitations deserve mention. The first limitation of the study is the lack of a representative group of at-risk pregnant women. The inclusion/exclusion criteria precluded some at-risk pregnant women from participating in the study as women with psychopathological disorders or women with a history of mental disorders or adverse experiences during the past years. Several studies detected that a history of mental health problems or adverse experiences prior to pregnancy is associated with a decreased quality of mother-fetus interaction (Aktar et al., 2019; Smorti et al., 2020). Thus, further studies need to be carried out because the history of the pregnant women may interfere with biological and psychological mechanisms underpinning the mother-infant bond. Second, there is the risk of a memory bias, since the PBI is a measure that examines the perception of the past. In addition, limitations of the use of self-administered questionnaires like the PBI compared to other forms of assessment such as interviews, semi-structured interviews, or grids should be considered. Third, we did not have data on additional factors such as the sex of the infants, resilience and coping strategies of parents, and social support of pregnant women that might have an impact on the prenatal attachment process. Four, due to the cross-sectional design of the study, we cannot directly draw conclusions about cause and effect. Still, to our knowledge, the current study is the first report to include measures of parental bonding, psychological distress, and maternal-fetal attachment during pregnancy. An additional uncontrolled factor is the paternal-fetal attachment. Previous studies revealed that partners have lower fetal attachment scores than pregnant women (Dayton et al., 2019), thus clinical perinatal psychologists, midwives, and nurses have a great responsibility in terms of assessing and promoting attachment behaviors also in partners.

## Conclusion

Intergenerational transmission of parenting has been documented in different samples population studies and countries. However, previous studies have mainly focused on the mother-child attachment bond after birth. This study expands our knowledge on the effects of a woman’s perceived bond in relation to her parental figures during childhood and adolescence on the prenatal attachment process in a large population cohort of pregnant women. The results of this study indicate that maternal and paternal care parenting may affect the maternal-fetal bonding of the expectant mother positively. This heightens the hypothesis that parenting support and treatment may have advantages that are realized across generations. Further, by determining mothers’ attachment style and the status of maternal-fetal attachment during pregnancy, in time interventions and education could be planned for improving these interactions and consequently improve the mother-child attachment during and after pregnancy.

## Data availability statement

Dataset will be shared in accordance with explicit external requirements. Requests to access these datasets should be directed to francesco.craig@unical.it.

## Author contributions

FC substantial contributions to the conception, interpretation of data for the work, and revising it critically for important intellectual content, and drafting the work. MG substantial contributions to the conception, interpretation of data for the work, and revising it critically for important intellectual content. PT final approval of the version to be published. VM performed the statistical analysis, interpretation of data for the work, and drafting the work. JC design of the work and drafting the work. AA and IM design of the work and acquisition of data. FT revising it critically for important intellectual content. ACe and ACo final approval of the version to be published. All authors contributed to the article and approved the submitted version.
